# INDIRECT EFFECT OF PARTICIPATION ON THE ASSOCIATION BETWEEN FUNCTIONAL VISION AND SELF-RATED HEALTH OF OLDER ADULTS

**DOI:** 10.1093/geroni/igz038.534

**Published:** 2019-11-08

**Authors:** Bernard A Steinman, Casandra Mittlieder

**Affiliations:** University of Wyoming, Laramie, Wyoming, United States

## Abstract

Older adults with visual impairments experience barriers to participation that could impact their own health and wellbeing. Participation limitations that are associated with poor vision and unsupportive environments have been associated with objective health outcomes, including chronic disease and secondary outcomes such as fall injuries. In this study, we assessed the association between functional vision impairment and self-rated health (SRH). We also tested the mediating role of participation in that relationship. We conducted analysis with covariates representing six domains included in the International Classification of Functioning, Disability, and Health (ICF). We computed ordinal logistic regression models using two waves (2011 and 2016) of the National Health and Aging Trends Study (NHATS). Vision status was assessed via self-reported measures of visual functioning (e.g., ability to recognize someone across the street). “Participation” was operationalized using 6 items that assessed participation in social and community-based activities (e.g., visiting friends, participating in classes). SRH (measured in 2016) was assessed using a single item asking participants to rate their health on a scale ranging from excellent to poor. We assessed key relationships while holding ICF’s other health dimensions constant. Functional vision status was statistically associated with SRH in models containing all covariates. Participation variables reduced but did not eliminate the effects of vision, suggesting a partial mediating effect—that is, part of the association between vision and SRH was explained by participation factors. These results point to the importance of developing community support and reducing barriers to participation by older adults with functional vision impairments.

